# Approaches to stereoselective 1,1'-glycosylation

**DOI:** 10.3762/bjoc.21.133

**Published:** 2025-08-27

**Authors:** Daniele Zucchetta, Alla Zamyatina

**Affiliations:** 1 Department of Natural Sciences and Sustainable Resources, Institute of Organic Chemistry, BOKU University, 1190 Vienna, Austria

**Keywords:** carbohydrates, chemical glycosylation

## Abstract

Nonreducing disaccharides are prevalent in non-mammalian glycans and glycolipids, serving as pivotal structural components in mycobacterial glycans, microbial oligosaccharide and nucleoside antibiotics, as well as biologically active mimetics of bacterial pathogen-associated molecular patterns (PAMPs). As integral components of PAMPs, 1,1′-linked disaccharide-containing biomolecules play important roles in host–pathogen interactions, cellular signaling, and pathogenesis. Accessing complex biomolecules containing nonreducing disaccharides is often hindered by difficulties in isolating them from natural sources, which can result in impure or degraded products, particularly when sensitive functional groups are involved. Consequently, approaches to 1,1′-glycosylation for the synthesis of nonreducing disaccharides with defined anomeric configurations are essential for the development of 1,1′-disaccharide-containing biomolecules used in vaccine research, as well as for therapeutic and diagnostic applications. The assembly of nonreducing 1,1′-linked disaccharides presents greater challenges than conventional chemical glycosylation due to the need for simultaneous control of stereochemistry at two anomeric centers. The structural complexity of natural biomolecules entailing 1,1′-disaccharides, which feature asymmetrically distributed functional groups across their two pyranose rings, emphasizes the importance of robust, stereoselective synthetic strategies capable of producing fully orthogonally protected 1,1′-linked sugars suitable for selective chemical modification. This review highlights recent advances in 1,1′-glycosylation and provides an overview of selected glycosylation strategies, including those aimed at forming α,β-, β,β-, and α,α-1,1′-glycosidic linkages. Particular emphasis is placed on the challenges of achieving stereoselectivity with lactol glycosyl acceptors, which commonly exist as mixtures of anomers and are therefore problematic to use in chemical glycosylation reactions.

## Introduction

Nonreducing disaccharides are abundant components of non-mammalian glycans and glycolipids found in fungi [1], nematodes [2], and bacteria such as *Salmonella* [3] and *Mycobacterium* (e.g., *M. tuberculosis, M. smegmatis*, etc.) [4–9]. They are the constituents of microbial oligosaccharide antibiotics (mostly produced by genus *Micromonospora* and *Streptomyces*) belonging to the orthosomycins family (such as evernimicin, avilamycin, etc.) [10–14] and the nucleoside antibiotic tunicamycin [15–17], etc. These molecules play essential roles in cell signaling, host–pathogen interactions, and pathogenesis and, being the constituent of pathogen-associated molecular patterns (PAMPs), can function as critical virulence factor [7,18–25]. The availability of complex biomolecules containing nonreducing disaccharides is limited, as their isolation from natural sources is challenging and often fails to ensure the homogeneity of the isolates or the integrity of biomolecules bearing labile functional groups. Therefore, synthetic access to nonreducing disaccharides with different anomeric configurations is crucial for obtaining complex 1,1'-disaccharide-containing biomolecules for use in vaccine research [22,26–29], or for therapeutic [4,10,30–32] and diagnostic [19,33–35] applications.

The glycosylation reaction for forming nonreducing (1,1'-linked) disaccharides is inherently more complex than traditional glycosylation protocols, due to the importance of controlling the stereochemistry at two anomeric centers simultaneously. Consequently, the 1,1'-glycosylation reaction traditionally presents challenges such as modest stereoselectivity, the formation of various diastereomeric by-products and moderate yields. The configuration of the glycosidic linkage (ββ, αα or βα) is the most important factor in choosing an appropriate glycosylation approach, alongside the specific requirements for functional or protecting groups that substitute the hydroxy- or amino functions of the monosaccharide components. Over the past decade, significant progress has been achieved in the stereoselective construction of 1,1'-glycosides using both conventional glycosylation methodologies and innovative, specialized approaches. While the synthesis of α,α-1,1'-linked nonreducing sugars was already comprehensively reviewed [36–37], the construction of β,β- and β,α-1,1'-glycosides has received noticeably less attention. This review provides an overview of the most recent developments in 1,1'-glycosylation, alongside a retrospective on specific glycosylation approaches, focusing particularly on the construction of α,β- and β,β-1,1'-glycosidic bonds in pyranoses. Special emphasis is placed on the challenges of controlling stereoselectivity on the side of the lactol glycosyl acceptor, as lactols typically exist as a mixture of anomers which complicates their use in the chemical glycosylation reactions.

Earlier approaches to constructing 1,1'-*O*-glycosidic bonds often relied on the use of simply and symmetrically protected (benzylated or acetylated) monosaccharide building blocks, enabling the synthesis of symmetrically functionalized nonreducing disaccharide-based molecules. However, most biologically active molecules that contain nonreducing sugars are characterized by the presence of non-symmetrically distributed functional groups, which requires the use of selectively orthogonally protected precursors [38–42]. This emphasizes the importance of reliable and reproducible approaches for the stereoselective synthesis of unsymmetrically orthogonally protected nonreducing disaccharides. While the desired anomeric selectivity on the side of the glycosyl donor can often be achieved by exploiting the participation effect of the neighboring protecting group [43–44], employing anomeric lactols as acceptors is more complex as lactols with the desired configuration are frequently difficult to obtain (e.g., through anomerization). In addition to the nature of the leaving group and promoter system, a variety of other factors commonly influences both the reaction rate and the stereochemical outcome of the glycosylation reaction, including the reactivity of the acceptor hydroxy group, the effect of remote protecting groups and a balance between the nucleophilicity of the acceptor and the reactivity of the donor molecules [45–48].

## Review

### Synthesis of β,α-1,1'-linked disaccharides

**Scheme 1:**
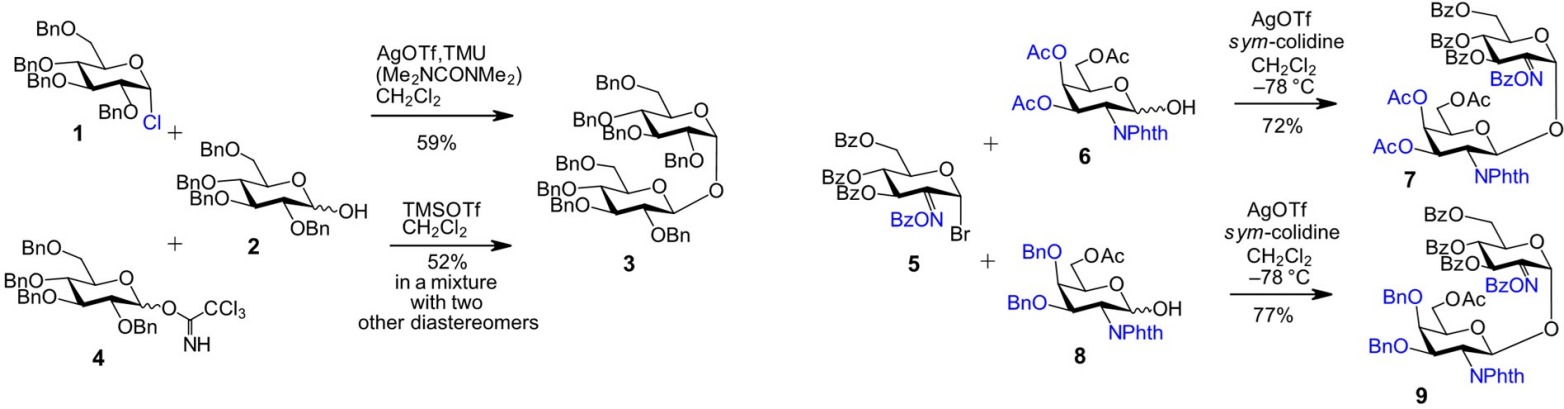
Application of chloride-, bromide-, and trichloroacetimidate donors in 1,1'-coupling reactions towards β,α-glycosides.

The assembly of β,α-1,1'-linked disaccharides has traditionally been carried out using perbenzylated monosaccharide building blocks and various types of glycosyl donors, including chlorides [49], bromides [50], and trichloroacetimidates [51–52]. However, the monosaccharide components were mostly fully benzylated or acetylated, so that the 1,1'-glycosylation reactions led to the formation of symmetric nonreducing products [53]. For instance, the tetrabenzylated chloride donor **1** was reacted with the tetrabenzylated glucose lactol acceptor **2**, resulting in the formation of the β,α-1,1-linked diglucoside **3** (Scheme 1) [49]. The application of a the 2,3,4,6-tetra-*O*-benzyl trichloroacetimidate donor **4** resulted in the same disaccharide product **3**, albeit as part of a mixture with other diastereomers [51].

The synthesis of neotrehalosamines related to the sugar moiety of the nucleoside antibiotic tunicamycin was performed using benzoylated 2-(benzoyloxyimino)-protected GlcN bromide donor **5**, which enabled 1,2-*cis* stereochemical control through the non-participating C-2 benzoyloxyimino group, leading to the formation of an α-glycoside on the donor side [54]. On the acceptor side, the β-configuration was favored due to steric hindrance from the C-2 phthalimido group in the triacetylated GalN-lactol acceptors **6** or 3,4-di-*O*-benzylated acceptors **8**, resulting in kinetically controlled formation of β,α-1,1'-neotrehalosamines **7** and **9**, respectively (Scheme 1) [38]. The stereochemical outcome was found to be independent of the initial anomeric configuration of the *N*-phthalimido-protected lactol acceptor, which was explained by kinetic control and a rapid anomerization in favor of the β-anomer [50].

**Scheme 2:**
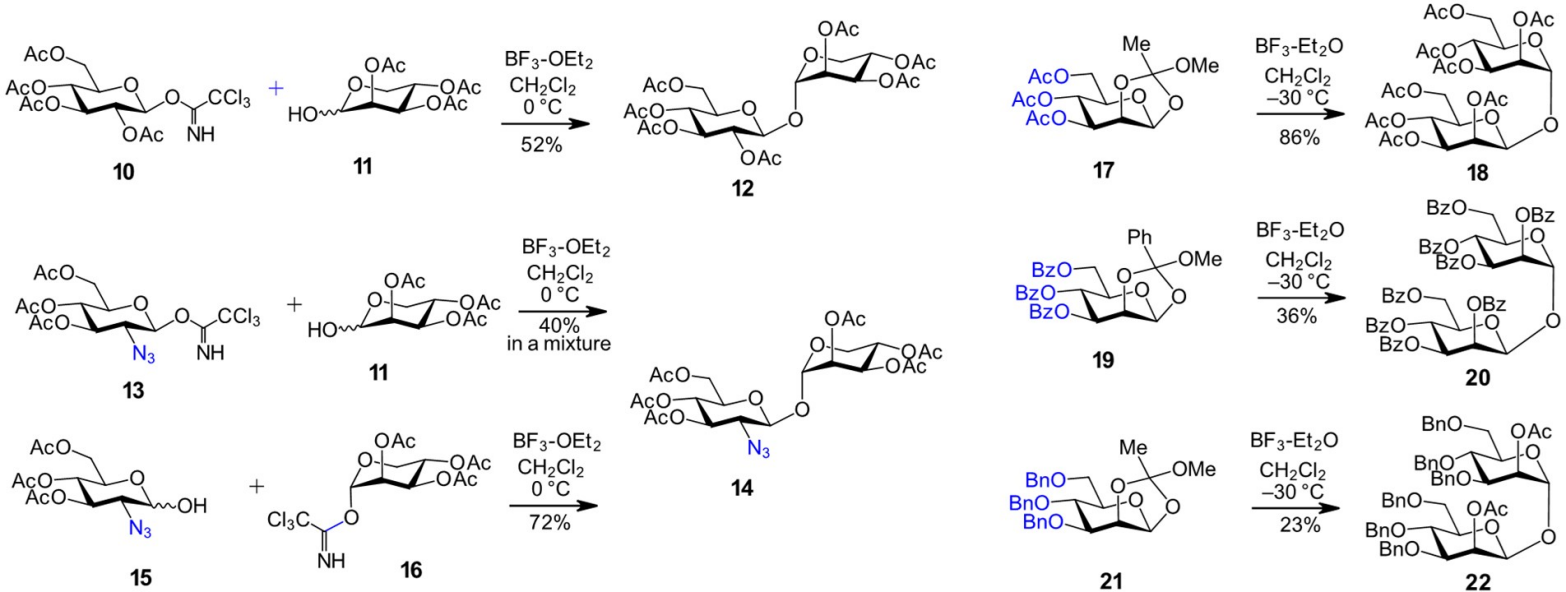
Application of trichloroacetimidates as donors in 1,1'-β,α coupling reactions and the use of 1,2-orthoacetates as a source of symmetrical β,α-1,1-disaccharides.

The applicability of trichloroacetimidates to the assembly of 1,1'-disaccharides was demonstrated in the synthesis of ʟ-lyxopyranosyl-β-ᴅ-glucopyranoside, a biosynthetic precursor of the FG ring system of avilamycin [52]. Peracetylated ᴅ-glucosyl trichloroacetimidate **10** was reacted with triacetylated ʟ**-**lyxose lactol acceptor **11** in the presence of boron trifluoride etherate as promoter to afford the β,α-1,1'-linked disaccharide **12** in 52% yield (Scheme 2). The electron-withdrawing effect of the 2-azido group in the structurally similar donor **13** led to a significant drop in efficiency and the formation of the desired disaccharide **14** along with several diastereomeric by-products [52]. In contrast, using a 2-azido-2-deoxy-Glc lactol **15** as the acceptor in combination with the ʟ-lyxopyranosyl donor **16** resulted in the exclusive formation of the β,α-linked product **14** (Scheme 2).

Symmetrically protected α,β-1,1'-dimannosides **18**, **20**, and **22** were readily prepared by self-condensation of the corresponding acetylated methyl 1,2-orthoacetates using BF_3_·OEt_2_ as a promoter [55]. While the 1,2-orthoester functionality in the glycosyl donor **17** directed the 1,2-*trans* selectivity towards the formation of an α-mannosidic linkage, the anomeric β-configuration on the glycosyl acceptor side was “retained” from the 1,2-orthoester progenitor. The nature of the protecting groups proved to be critical for the glycosylation outcome: the use of bulkier benzoyl esters, as in the phenyl 1,2-orthoester **19**, led to a lower yield (36%) of the 1,1'-linked product **20** (Scheme 2). Switching from “disarming” ester to “arming” benzyl protecting groups, as in the donor **21**, enhanced the activation of the glycosidic center, resulting in the formation of a substantial amount of the methyl glycoside by-product and a reduced yield of the 1,1'-disaccharide **22**.

**Scheme 3:**
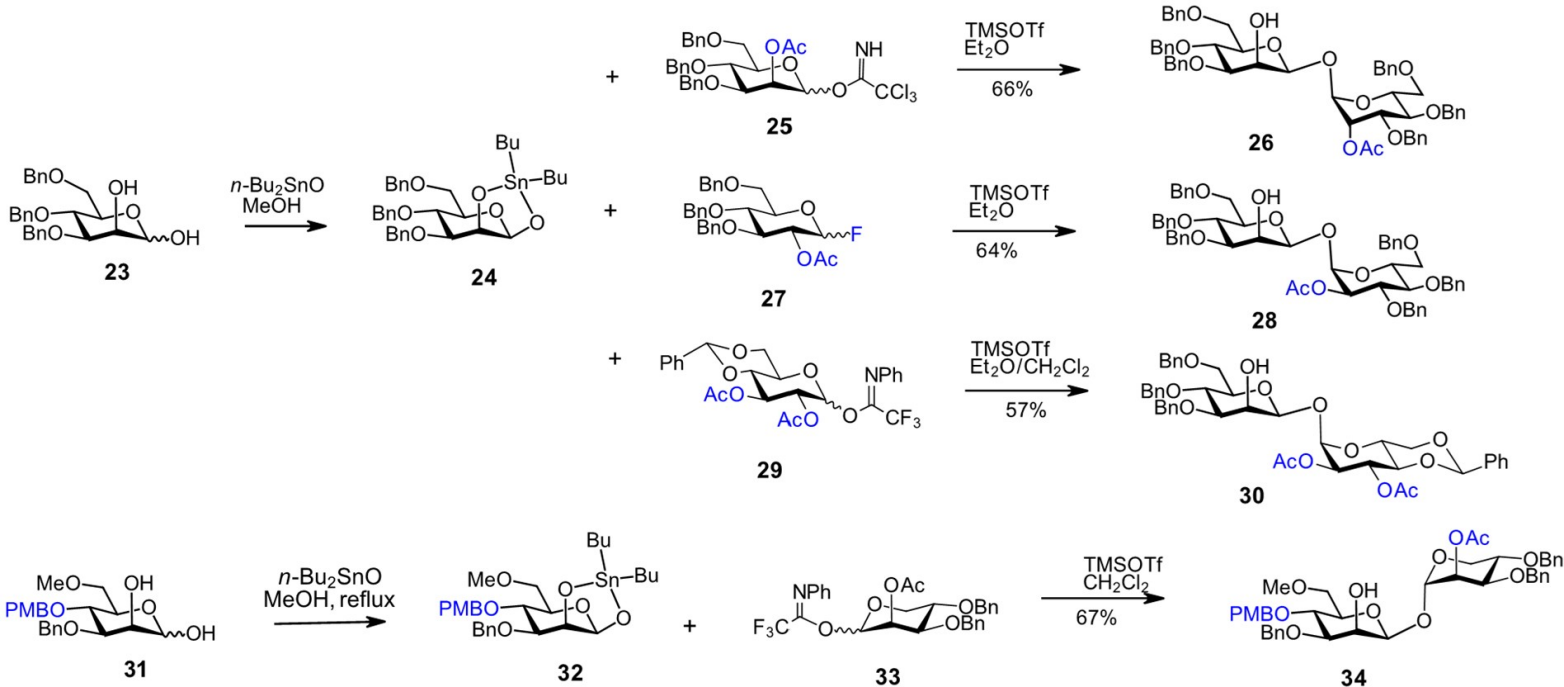
The β-anomeric configuration in the lactol acceptors can be trapped and fixed within the five-membered ring of the tin-acetal.

Securing the anomeric configuration of the lactol acceptor is particularly challenging when the desired form is not thermodynamically favored, as is the case with β-mannose derivatives. A refined strategy employing cyclic stannanes to lock the anomeric hydroxy group of a mannose-derived lactol in the equatorial orientation has proven both efficient and reliable [56–58]. The axial C2-OH group in the tribenzylated Man-derived lactol **23** was utilized to trap the anomeric oxygen in the β-configuration via a five-membered stannane ring in **24** (Scheme 3). Subsequent glycosylation with the trichloroacetimidate donor **25** led to the stereoselective formation of β,α-1,1'-dimannoside **26** in 66% yield [57]. The procedure also demonstrated excellent stereoselectivity with the fluoride donor **27**, resulting in the formation of β-mannosyl-α-glucoside **28**. Furthermore, the use of acetyl and temporary 4,6-*O*-benzylidene protecting groups, as in the imidate donor **29**, instead of benzyl ethers, was fully compatible and resulted in the exclusive formation of β-mannosyl-α-glucoside **30** [57]. This approach was successfully applied to the synthesis of a nonreducing disaccharide moiety of evernimicin, an octasaccharide antibiotic that exhibits activity against multidrug-resistant *Streptococci* and *Staphylococci* including *S. aureus* via a unique mechanism involving binding to an exclusive site on the bacterial ribosome [10,59]. Accordingly, the β-anomeric configuration in the 3-*O*-*p*-methoxybenzyl-protected mannose lactol **31** was fixed by the formation of a five-membered tin-acetal ring in **32**, which was subsequently glycosylated by the ʟ-lyxose imidate donor **33**, containing a participating 1,2-*trans*-directing 2-*O*-Ac group to produce the β,α-1,1'-disaccharide **34** corresponding to the F and G rings of evernimicin (Scheme 3) [58].

Diarylborinic acids have been shown to provide exclusive catalytic performance in the site-selective monofunctionalization of various 1,2- and 1,3-diols [60], as well as in the regioselective glycosylation of polyhydroxyglycosyl acceptors via base-promoted deprotonation of a specific hydroxy group involved in the formation of a borate complex [61]. Diarylborinic acids have also been exploited for stereoselective 1,1'-glycosylations through the formation of a 1-*O*-monoborinate ester, resulting from the complexation of a 1,2-dihydroxyglycosyl acceptor with a diarylborinic acid derivative. Concurrent coordination of the boron center with the remaining C2-OH group increases its acidity, thereby generating in situ an acidic catalyst for the activation of the glycosyl donor [62–63].

**Scheme 4:**
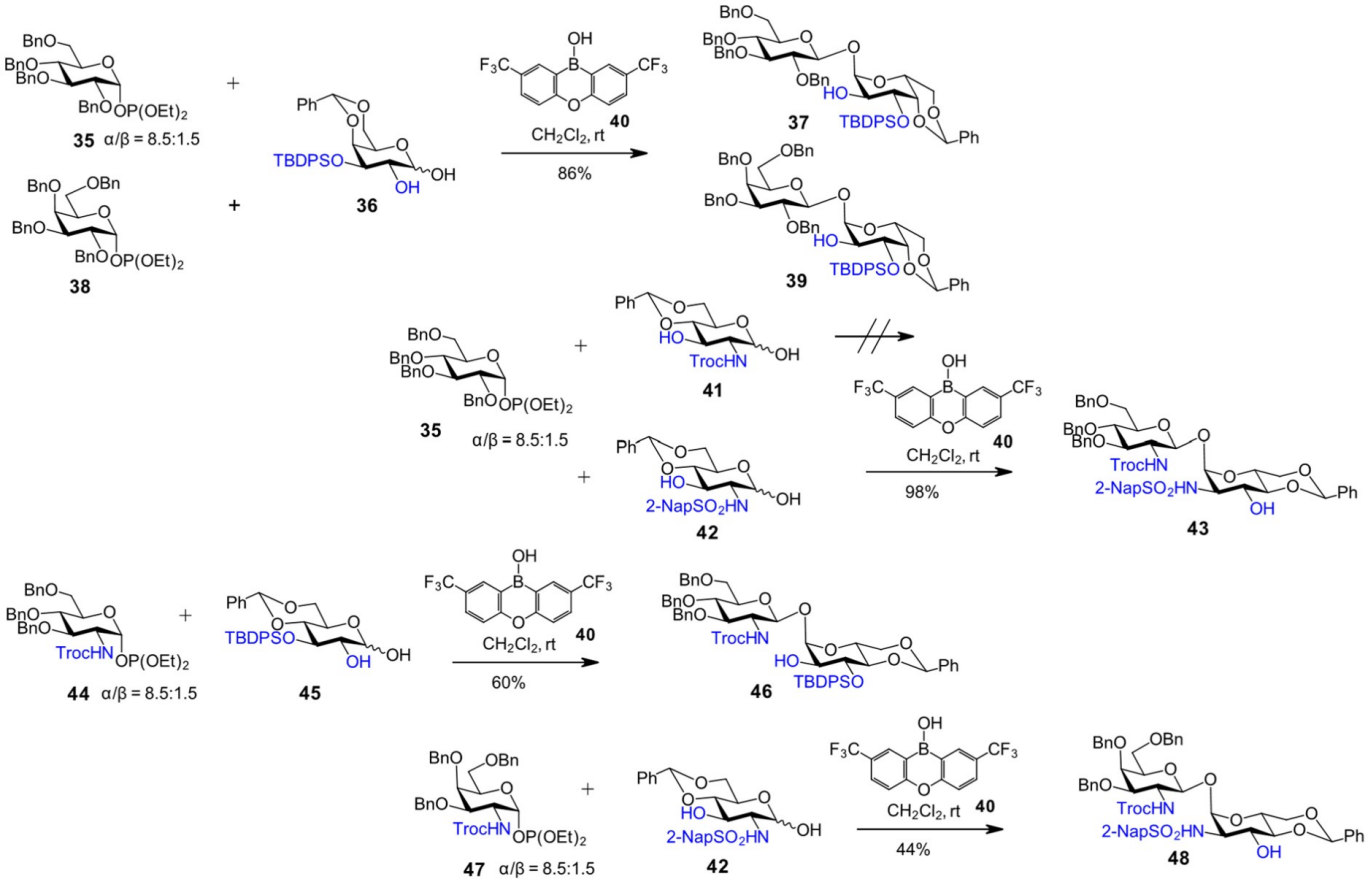
Diarylborinic acid-promoted β,α-1,1' glycosylation.

In this approach, the use of glycosyl phosphites of *gluco*- (**35**) and *galacto*- (**38**) configuration as donors, in combination with the orthogonally protected diol acceptor **36**, proved to deliver optimal stereoselectivity and the highest yields in the reaction promoted by bistrifluoromethylated tricyclic borinic acid **40**, affording β,α-1,1'-linked disaccharides **37** [63] and **39** [62], respectively, in 86% yield (Scheme 4). The nature of the 2*N*-protecting group on GlcN- and GalN-derived lactol acceptors proved crucial for the success of borinic acid-promoted glycosylation, as the 2*N*-Troc-protected acceptor **41** failed to participate in the reaction. In contrast, the 2*N*-naphthalenesulfonyl (NapSO₂)-protected glucosamine-derived lactol **42** afforded the corresponding 1,1'-linked diglucosamine **43** in almost quantitative yield in the borinic acid-promoted glycosylation with the phosphite donor **35** (Scheme 4). This outcome was attributed to the involvement of the NH group in complex formation with the borinic acid catalyst, suggesting that its sufficient acidity is key to the success of the reaction. Using the 2*N*-Troc-protected GlcN phosphite **44** as the donor and the 4,6-*O*-benzylidene acetal-protected 1,2-diol **45** as the acceptor afforded the β,α-disaccharide **46** with high stereoselectivity under borinic acid catalysis [62]. In addition, 2-amino-2-deoxy-1,3-diols were successfully employed as glycosyl acceptors, as exemplified by the use of GlcN-derived lactol **42**, which was reacted with the 2*N*-Troc-protected GalN phosphite donor **47** in the presence of borinic acid catalyst to give the β,α-1,1'-linked disaccharide **48** (Scheme 4) [63].

**Scheme 5:**
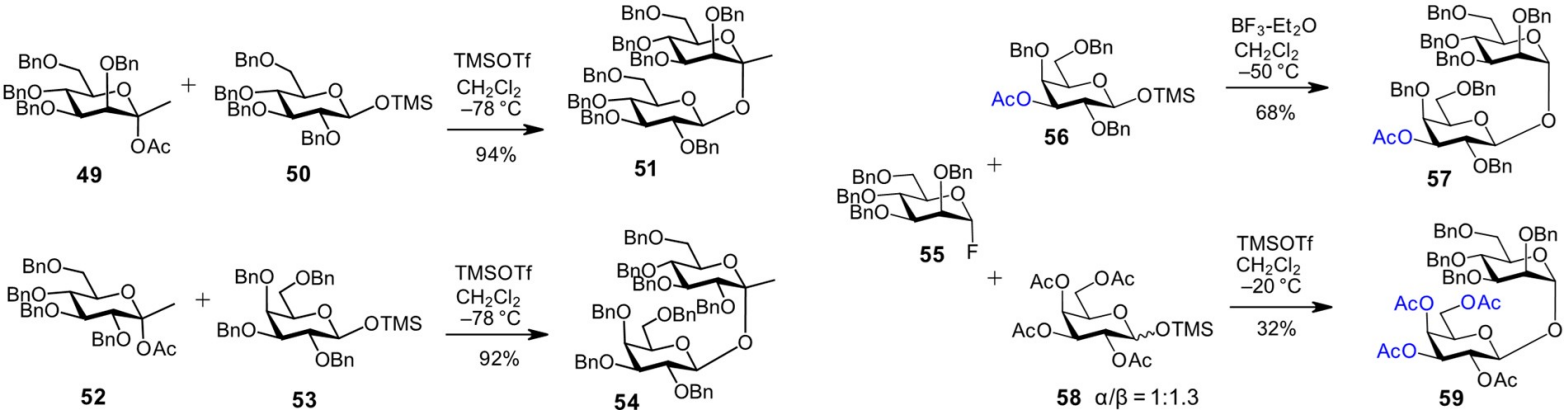
The anomeric configuration in the lactol acceptor can be trapped in the form of a TMS-glycoside.

The desired anomeric configuration of the lactol acceptor can also be stabilized through covalent modification; for example, in the form of a trimethylsilyl glycoside. A successful 1,1'-glycosylation using benzyl-protected monosaccharide precursors and 1-*O*-TMS-protected acceptors is exemplified by the synthesis of ketoside analogs of pseudo-trehalose. In this context, the *manno*-hept-2-ulose **49** and *gluco*-hept-2-ulose **52** were used as glycosyl donors, while 1-*O*-TMS-protected β-glucoside **50** or β-galactoside **53** served as glycosyl acceptors (Scheme 5) [64]. Since silyl glycosides can also act as glycosyl donors [65–66], care must be taken, and glycosylation conditions optimized to avoid the formation of oxocarbenium ions from TMS-glycosides intended to function as glycosyl acceptors. In this case, the ketoses **49** and **52** formed the oxocarbenium ions more readily due to stabilization by their alkyl substituents, thereby minimizing the likelihood of oxocarbenium ion formation from the silyl aldoside acceptors **50** and **53** (Scheme 5). Nucleophilic attack by 1-*O*-trimethylsilyl β-pyranosides **50** or **53** on the α-face of the oxocarbenium ions afforded the corresponding β-ketopranosyl α-aldopyranosides **51** and **54**, respectively [64].

The use of TMS-stabilized lactol acceptors also found application in the synthesis of an E-selectin inhibitor. The tribenzylated TMS-β-galactose acceptor **56** was reacted with the tetrabenzylated mannosyl fluoride donor **55**, ensuring high stereoselectivity in the formation of the α,β-1,1'-conjugated product **57**, a precursor of the E-selectin inhibitor (Scheme 5) [39–40]. However, when the benzyl protecting groups were replaced with acetyl groups, as in the case of the tetraacetylated Gal-derived acceptor **58**, the anomeric hydroxy group could only be partially trapped in the β-configuration (α/β = 1:1.3), leading to a loss of stereoselectivity and affording the α/β-linked product **59** in 32% yield [40].

The stereodirecting effect of the *O*-picoloyl protecting group at remote position on the pyranose ring (C3–OH, C4–OH, or C6–OH) in glycosyl donors has been known to induce high facial selectivity in glycosylations with various types of nucleophiles [67–68]. This remarkable stereoselectivity has been attributed to intermolecular hydrogen bonding, which forms a transient tether between the glycosyl donor and acceptor, thereby guiding the reaction towards a preferred stereochemical outcome, a mechanism known as HAD (H-bond-mediated aglycone delivery) [68]. The picoloyl protecting group at the remote C4–OH position has also been successfully employed to stabilize the anomeric configuration in 2,3,6-tri-*O*-benzylated TMS-glycoside acceptors [69]. This unusual stability of the anomeric configuration has been explained by the formation of a picolinium adduct, resulting from the trapping of the acidic by-product TMSOTf by the pyridine moiety of the picoloyl group during glycosylation [69]. The resulting intermediate, a picolinium-stabilized TMS-glycoside, exhibits high configurational stability due to the strong electron-withdrawing effect of the picolinium substituent, effectively preventing anomerization or self-condensation.

**Scheme 6:**
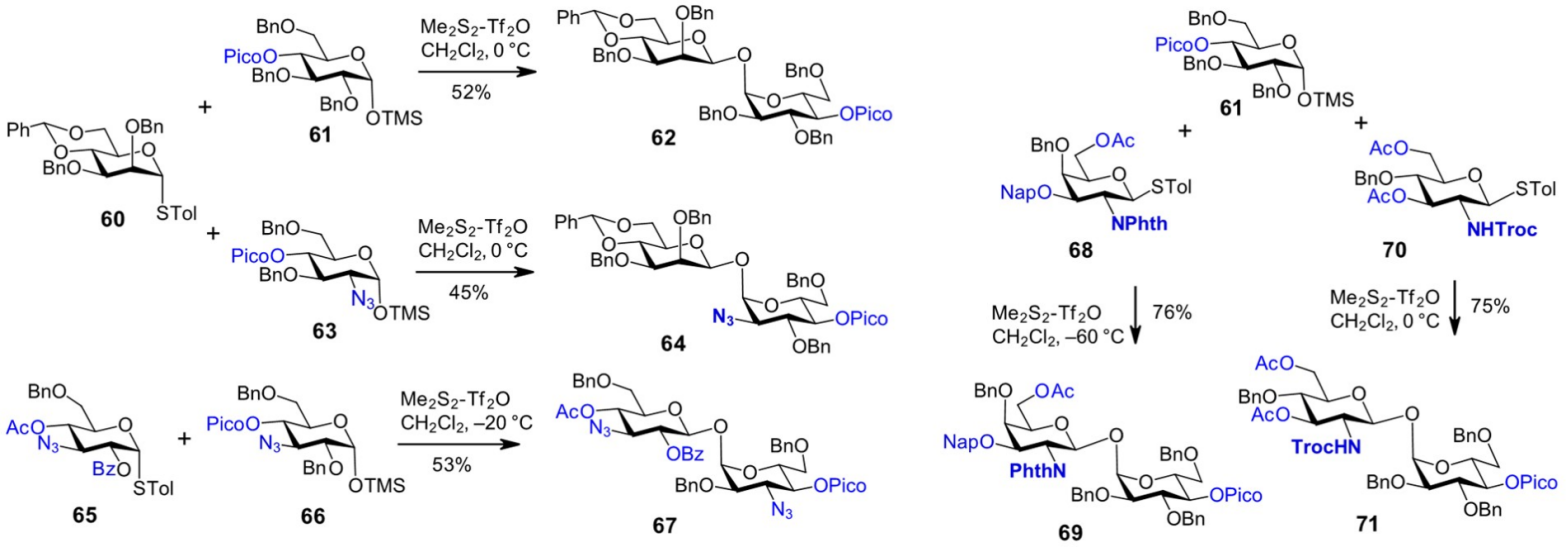
The anomeric configuration in the lactol acceptor can be trapped in form of a 1-*O*-TMS-glycoside that is additionally stabilized by a remote picoloyl group.

Good stereoselectivity and yields were achieved in the glycosylation reactions of 4-*O*-picoloyl-protected TMS-α-glycoside acceptors with stereodirecting thioglycoside donors of *gluco*-, *galacto*-, and *manno*-configuration, using a sulfonium-type promoter generated in situ from dimethyl disulfide (Me_2_S_2_) and triflic anhydride (Tf_2_O) [66,70]. The use of common acid scavengers, such as DTBP (di-*tert*-butylmethylpyridine), was not recommended, as glycosylation reactions involving TMS-glycoside acceptors require consistently acidic conditions, which places special demands on the acid stability of the protecting groups employed. This approach enabled the synthesis of β,α-1,1'-linked β-mannosyl-α-glucoside **62** and its 2-azido-2-deoxy-derivative **64**, using the 4,6-*O*-benzylidene-protected mannose-derived thioglycoside donor **60** and TMS-1-*O*-glycosides **61** and **63**, respectively (Scheme 6) [69].

Similarly, the β,α-1,1'-glucoside **67**, bearing two symmetrically positioned 3-azido groups, was obtained from TMS-α-glucoside **66** and the β-directing thioglycoside donor **65**, bearing electron-withdrawing protecting groups (OAc and OBz) at different positions (Scheme 6) [71]. Nonreducing disaccharides containing 2-amino-2-deoxypyranoses were also synthesized using this approach. The 1,2-*trans*-directing effect of the 2*N*-phthalyl and 2*N*-Troc groups in thioglycosides **68** and **70** enabled the stereospecific synthesis of β,α-linked disaccharides **69** and **71**, respectively, upon reaction with the TMS-α-Glc acceptor **61** [69]. The relatively high reactivity values (RRVs) reported for 2*N*-Troc-protected thioglycosides suggest that introducing an electron-withdrawing substituent should not substantially diminish the reactivity of *N*-Troc-protected GlcN-derived donors [72]. Indeed, the presence of an electron-withdrawing group at position 3 of the GlcN-derived donor **70** merely required an elevated reaction temperature (0 °C), in contrast to the conditions used for the 3-*O*-Nap-protected GalN-based donor **68** (reaction temperature −60 °C).

Permanent ether-type protecting groups, such as benzyl, or those that require harsh acidic or basic conditions for removal, are often incompatible with the labile functional groups present in complex biomolecules derived from synthetic nonreducing disaccharides. To address this shortcoming, it is essential to use monosaccharide building blocks bearing temporary protecting groups that can be selectively and sequentially removed under mild conditions. Such an approach ensures compatibility with sensitive functionalities and enables the effective incorporation of 1,1'-disaccharides into multifunctionalized biomolecules. 1,1'-Glycosylation using variably orthogonally protected monosaccharide donors and lactol acceptors presents additional challenges due to the well-known influence of protecting groups on the stereoselectivity of glycoside formation [73].

**Scheme 7:**
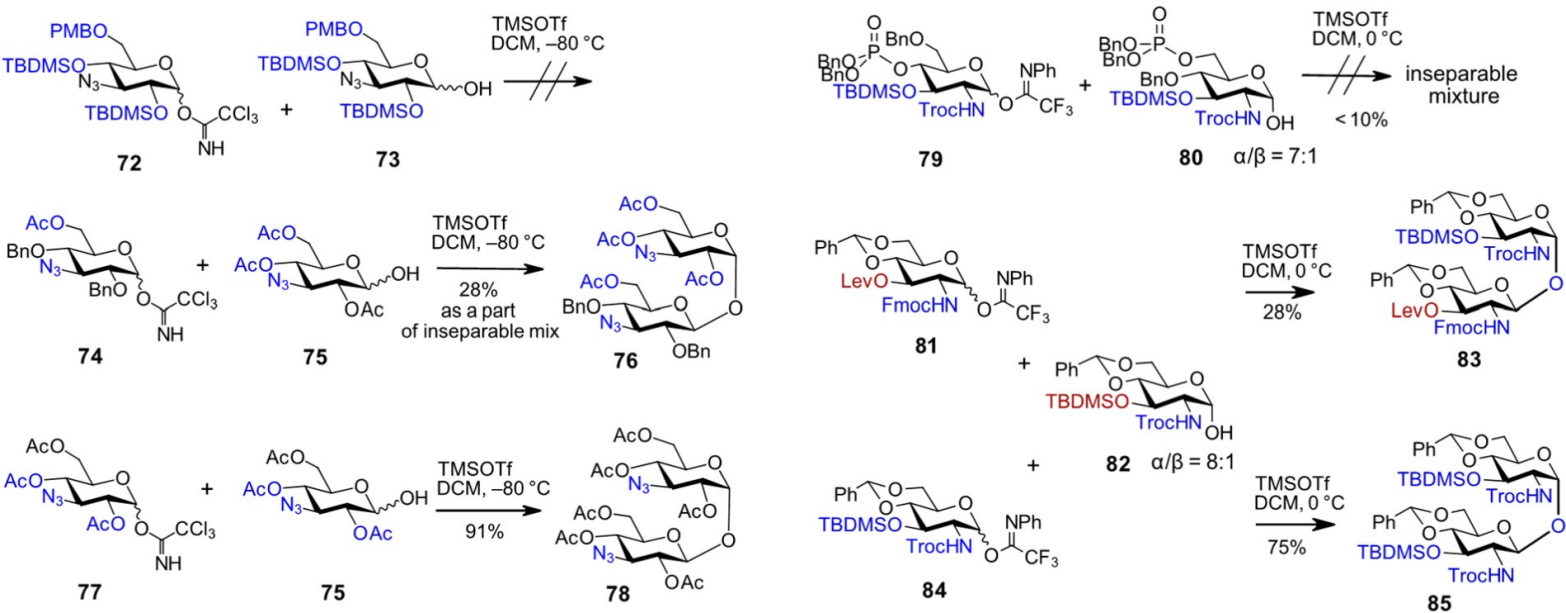
Influence of remote protecting groups on the stereoselectivity and efficiency of 1,1'-β,α bond formation.

An instructive example of employing differently protected, armed and disarmed donor–acceptor pairs in a β,α-1,1'-glycosylation reaction is demonstrated in the synthesis of 3,3′-neotrehalosadiamine – an antibiotic candidate isolated from *Bacillus pumilus* and *Bacillus circulans*, with putative activity against *S. aureus* and *K. pneumoniae* [74–75]. The application of variably protected 3-azido-3-deoxy-glucose-derived donor **72** and lactol acceptor **73**, both allegedly armed due to the presence of multiple electron-donating bulky substituents, did not lead to product formation in the TMSOTf-promoted glycosylation reaction (Scheme 7). A combination of the partially armed donor **74** with a disarmed acceptor **75** resulted in an inseparable mixture containing the β,α-linked disaccharide **76**, along with multiple by-products. In contrast, coupling of the disarmed donor–acceptor pair **77** and **75** afforded the desired β,α-1,1-disaccharide **78** in excellent yield [41].

Another example of protecting groups-mediated control of the stereoselectivity in β,α-1,1'-glycosylation demonstrated, that bulky electron-rich groups on both acceptor and donor molecules are disadvantageous, whereas a balanced combination of electron-donating and electron-withdrawing substituents leads to improved yields and stereoselectivities. Accordingly, the combination of GlcN-derived lactol **80** and the 2*N*-Troc-protected GlcN imidate donor **79**, both bearing multiple bulky substituents such as dibenzyl phosphate, TBDMS, and Troc protecting groups, resulted in a complex, inseparable mixture of products (Scheme 7) [76]. The use of a torsional disarming 4,6-*O*-benzylidene acetal protecting group on both the *N*-phenytrifluoroacetimidate donor **81** and the lactol acceptor **82** led to a moderate improvement in glycosylation efficiency. A significant advancement was observed upon increasing the reactivity of the imidate donor by replacing the electron-withdrawing 3-*O*-levulinoyl group with a TBDMS protecting group in **84**, which ultimately afforded the β,α-1,1'-disaccharide **85** in 75% yield [76].

These studies narratively demonstrate that the combination of variable temporary protecting and functional groups on glycosylation partners involved in 1,1'-bond formation can lead to unfavorable outcomes, including either the failure to form the desired 1,1'-disaccharide or the generation of multiple by-products. In addition to the stereodirecting participating protecting group on the glycosyl donor, the influence of remote protecting groups on donor reactivity and acceptor nucleophilicity should not be underestimated. This necessitates careful optimization of reaction conditions and the rational selection of compatible donor–acceptor pairs [77].

Protecting groups can also be used to fix a specific geometry in the glycosyl donor that favors the glycosyl acceptor approaching from only one possible side during the glycosylation reaction, as demonstrated in the stereospecific synthesis of β-fructofuranosyl disaccharides (e.g., sucrose) [78] which are also components of esterified sucrose derivatives in plants [79–81]. In this case, the carboxymethyl group in the 2-thioethyl fructofuranoside was locked by an intramolecular bridge formed by the TIPDS group to the C4–OH [1,4-*O*-(1,1,3,3-tetraisopropyldisiloxane-1,3-diyl) bridge], resulting in a β-directing fructofuranosyl donor [78,82–83]. Consequently, the Glc-lactol acceptor could approach the transient oxocabenium ion only from the β-face, thereby favoring the stereospecific formation of the α-glucopyranosyl-β-fructofuranoside in the DMTST/DTBMP-promoted glycosylation [78]. Other known chemical syntheses of sucrose and its derivatives include the use of β-ᴅ-psicofuranosyl donors [84–85] and the intracellular aglycon delivery (IAD) strategy [86].

Electron-withdrawing groups, often used for the temporary protection of hydroxy functions in pyranoses, can significantly reduce the nucleophilicity of both glycosyl donors and lactol acceptors. This often necessitates specific conditions, such as elevated reaction temperatures (above 0 °C), which are frequently incompatible with the use of thioglycosides as glycosyl donors [87]. Unlike thioglycosides, which typically require equimolar amounts of thiophilic promoters and reduced reaction temperatures (−20 to −60 °C), *N*-phenyl trifluoroacetimidate (PTFA) donors are sufficiently stable to be used in glycosylation reactions at 0 °C or ambient temperature, while remaining reactive enough to glycosylate conformationally hindered nucleophiles in the presence of catalytic TMSOTf as a promoter [88–89]. These considerations guided the synthesis of a series of differently orthogonally protected 1,1'-diglucosamines.

**Scheme 8:**
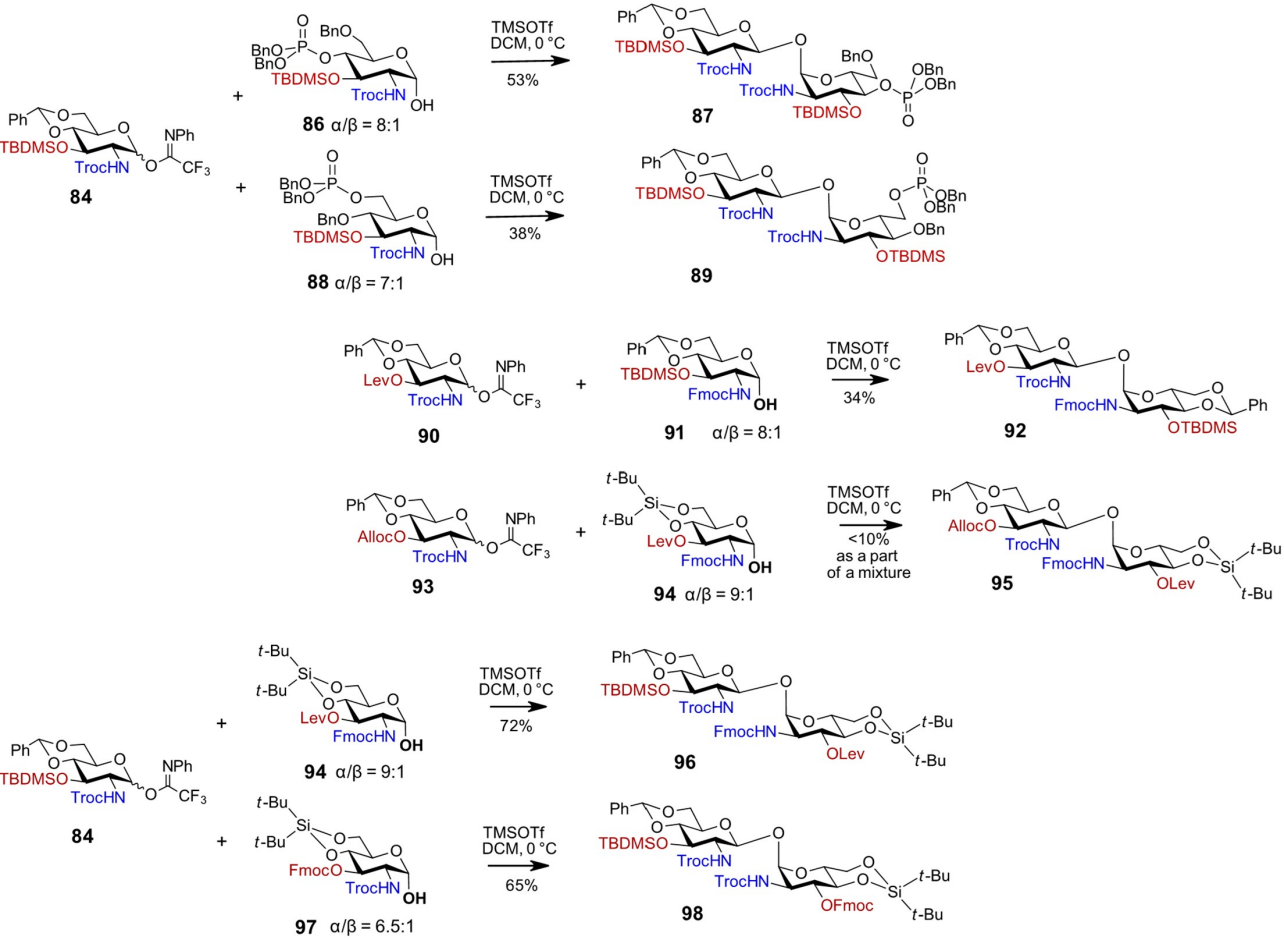
Synthesis of non-symmetrically fully orthogonally protected β,α-1,1' diglucosamines.

The choice of temporary neighboring protecting groups in the 2-amino-2-deoxypyranose series is rather limited, with *N*-carbamates (e.g., *N*-Troc and *N*-Fmoc) being well known for their strong 1,2-*trans*-stereodirecting effect [44,90–91]. On the other hand, the use of *N*-Troc and *N*-Fmoc as protecting groups in GlcN-based lactol acceptors offers the advantage of stabilizing the α-configuration via an intramolecular hydrogen bond between the axial anomeric hydroxy group and the 2*N*-carbamate, leading to a significant shift towards the α-anomer (α/β = 7:1 to 9:1) [76]. Accordingly, the β,α-1,1'-diglucosamine **87** was synthesized via glycosylation of the conformationally armed 2*N*-Troc-protected lactol acceptor **86** with the torsionally locked glycosyl imidate donor **84** (Scheme 8). Enhancing the acceptor reactivity by introducing a benzyl group at the C4–OH and relocating the phosphate group from position 4 to 6, as in GlcN-lactol **88**, resulted in a decreased yield of the β,α-1,1'-linked product **89**.

Despite the high prevalence of the α-anomer in the acceptors **86** and **88** (α/β = 8:1 and 7:1, respectively), the β,β-configured by-products were also formed, which was attributed to rapid anomerization of the lactol acceptor to the more reactive β-anomer during glycosylation. To minimize anomerization, the lactol acceptor was locked in a ^4^*C*_1_ conformation using a 4,6-*O*-benzylidene acetal group. The 4,6-*O*-benzylidene acetal exerts both torsional and electronic disarming effects, influencing the reactivity of glycosyl donors and the nucleophilicity of glycosyl acceptors, thereby improving the stereoselectivity of glycosylation [92–94]. In general, 4,6-*O*-cyclic protecting groups impose a torsional constraint that stabilizes the thermodynamically preferred α-configuration of the lactol, while also electronically deactivating the β-anomer, making it less reactive under glycosylation conditions. Consistent with this, the glycosylation of the conformationally locked, α-anomer-rich lactol acceptor **91** (α/β = 8:1) with the 4,6-*O*-benzylidene-3-*O*-levulinoyl-2-*N-*Troc-protected donor **90** resulted in the exclusive formation of the orthogonally protected β,α-1,1'-linked disaccharide **92** (Scheme 8) [95]. The moderate isolated yield was attributed to difficulties in separating the product from co-migrating monosaccharide by-products.

To synthesize fully orthogonally protected 1,1'-linked diglucosamines, the influence of specific combinations of protecting groups on the nucleophilicity of the lactol acceptor and the reactivity of the glycosyl donor was taken into consideration. To achieve a full desymmetrization of both pyranose moieties by additionally differentiating the protecting groups at C6–OH and C4–OH of each monosaccharide component, the cyclic 4,6-*O*-di-*tert*-butylsilylene (DTBS) group was installed as seen in the orthogonally protected lactol acceptor **94** (Scheme 8) [95]. The conformationally restricting cyclic DTBS group is less disarming compared to the 4,6-*O*-benzylidene acetal, thereby preserving the nucleophilicity of the lactol acceptor, while it is also known to limit the number of potential conformations of the pyranose ring during glycosylation [96–97]. However, the glycosylation reaction of the conformationally and electronically disarmed imidate donor **93** with 4,6-*O*-DTBS-protected α-lactol **94** (α/β = 9:1) afforded only trace amounts of the desired β,α-1,1'-product **95** as a part of a complex mixture, indicating mismatched reactivities of the donor–acceptor pair.

The presence of silyl groups on secondary hydroxy groups in pyranoses is known to exert a strong arming effect on the glycosidic center, with reactivity enhancements particularly evident when introduced at the C3–OH or C4–OH positions [98–100]. In this context, replacing the 3-*O*-Alloc group in trifluoroacetamide donor **93** with a 3-*O*-TBDMS group, as in compound **84**, significantly boosted donor reactivity. This more reactive donor was successfully employed in a TMSOTf-promoted glycosylation reaction with the orthogonally protected lactol acceptor **94**, affording the fully desymmetrized β,α-1,1'-diglucosamine **96** in an excellent yield [95]. Likewise, the coupling of donor **84** with 3-*O*-Fmoc-protected lactol acceptor **97** gave the orthogonally protected β,α-1,1'-disaccharide **98** as a single product (Scheme 8). It is notable that the 3-*O*-Fmoc group in lactol acceptor **97** (α/β = 6.5:1) had limited capacity to promote anomerization to the α-anomer, whereas the electron-withdrawing 3-*O*-levulinoyl group significantly enhanced the α-anomeric preference of the GlcN lactol acceptor **94** (α/β = 9:1). The slightly greater flexibility of the pyranose ring observed in DTBS-tethered pyranoses, relative to their benzylidene-protected counterparts, was considered beneficial for forming the conformationally strained β,α-1,1'-disaccharides [76,101].

### Synthesis of β,β-1,1'-linked disaccharides

One of the central challenges in β,β-1,1'-glycosylation consists in maintaining strict control over the β-anomeric configuration of the glycosyl lactol acceptor. This challenge has been effectively resolved by employing TMS-glycosides with stable configurations as the glycosyl acceptors by incorporating a picoloyl group positioned remotely from the anomeric center [69,71]. In glycosylation reactions that are promoted by dimethyl disulfide/triflic anhydride and use thioglycosyl donors, 4-*O*-picoloyl-protected 1-*O*-TMS-lactol acceptors have been shown to exhibit enhanced β-stereoselectivity, likely due to the formation of a transient picolinium adduct with the TMS-glycoside.

**Scheme 9:**
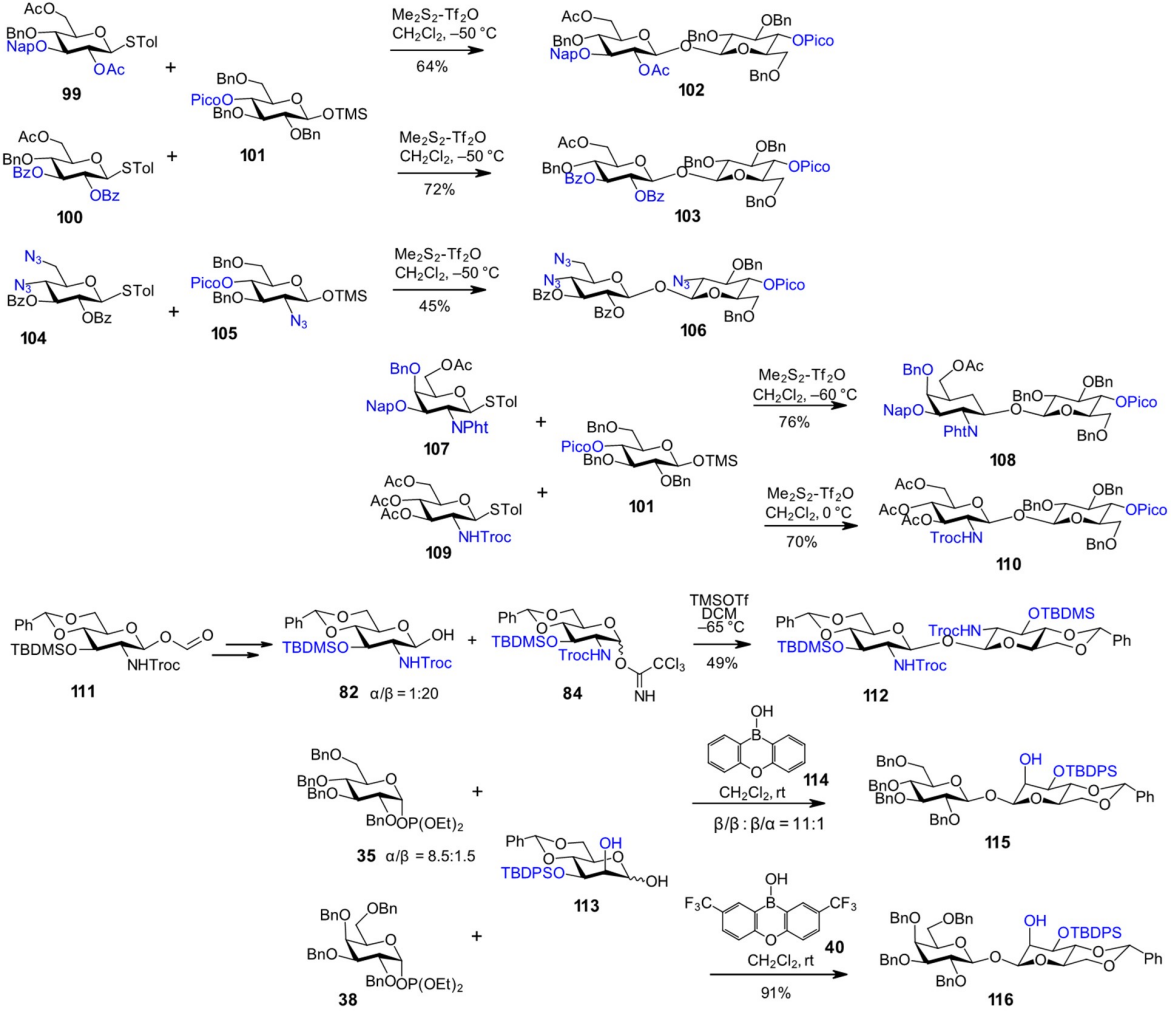
Synthesis of non-symmetric β,β-1,1'-linked disaccharides.

Tribenzylated 4-*O*-picoloyl-1-*O*-TMS-β-glycoside **101** demonstrated excellent stereoselectivity in glycosylation reactions with 1,2-*trans*-stereodirecting thioglycoside donors **99** and **100**, despite differences in the nature of protecting groups at the C3–OH [69]. The β,β-linked diglucosides **102** and **103**, featuring benzyl protecting groups on one Glc moiety and variable ether- and ester-type protecting groups on the other, were obtained in yields of 64% and 72%, respectively (Scheme 9). Synthesis of aminosugar-containing nonreducing disaccharides proved being more challenging, as demonstrated by the use of 2-azido-2-deoxy 1-*O*-TMS-glycoside **105** as acceptor [71]. The reaction of a disarmed 4,6-diazido-dideoxy donor **104** with 4-picoloyl-protected 1-*O*-TMS-β-glycoside **105** furnished the β,β-1,1'-linked disaccharide **106**, albeit with a lower yield. Alternatively, glycosylation reaction using 2*N*-Phth-protected GalN and 2*N*-Troc-protected GlcN donors **107** and **109**, respectively, and a common tribenzylated TMS-β-glycoside acceptor **101** afforded the glycosides βGalN(1↔1)βGlc **108** [71] and βGlcN(1↔1)βGlc **110** [69] with high stereoselectivity and yield (Scheme 9).

An alternative approach to β-configured GlcN-derived lactols has been proposed, based on a protocol for the traceless hydrolysis of β-GlcN-derived allyl glycosides with retention of the anomeric configuration [102]. Anomeric deallylation was achieved through an initial double-bond isomerization to the propenyl moiety, followed by oxidation of the glycosidic prop-1-enyl group to a formyl group, as in **111**, with subsequent basic anhydrous methanolysis yielding the β-configured GlcN-lactol **82** [103]. Glycosylation with the trichloroacetimidate donor **84** was carried out at a reduced temperature of −65 °C to suppress lactol anomerization, ultimately affording the β,β-configured 1,1'-diglucosamine **112**.

β,β-1,1'-Disaccharides were also synthesized using glycosyl organoboron catalysis and employing 1,2-diols as acceptors, while activation of the glycosyl donor was facilitated by the "acidic" C2–OH group of the borinic acid-complexed acceptor moiety [63]. For instance, the phosphite donor **35** was coupled with a Man-derived 1,2-diol acceptor **113** in the presence of a diarylborinic acid catalyst **114**, resulting in the formation of the βGlc(1↔1)βMan product **115** (Scheme 9). Similarly, the β,β-linked disaccharide **116** was synthesized using the same orthogonally protected 1,2-diol acceptor **113** and the tetrabenzylated GalN donor **38**, mediated by a modified borinic acid catalyst **40** [63].

Another glycosylation strategy that enabled access to fully orthogonally protected βGlcN(1↔1′)βGlcN disaccharides integrates well-established principles of 1,2-*trans* glycosylation for glycosyl donors, along with a carefully optimized selection of distinct protecting groups for lactol acceptors, focusing on defined electronic and torsional effects that influence their reactivity and anomeric preference. Among the various amino-protecting groups evaluated, only the 2-azido substituent was found to induce a conformational bias favoring β-configured lactols, rendering it the most appropriate choice for 2*N*-protection in GlcN-derived lactol acceptors. The electronic effects of remote protecting groups in the 2-N_3_-protected GlcN lactols were deliberately tuned to shift the anomeric ratio in favor of the β-anomer, although the inherent anomeric ratio typically ranged from α/β = 1:1 to 1:1.5, which indicates the potential formation of a diastereomeric mixture at the glycosyl acceptor side [95].

**Scheme 10:**
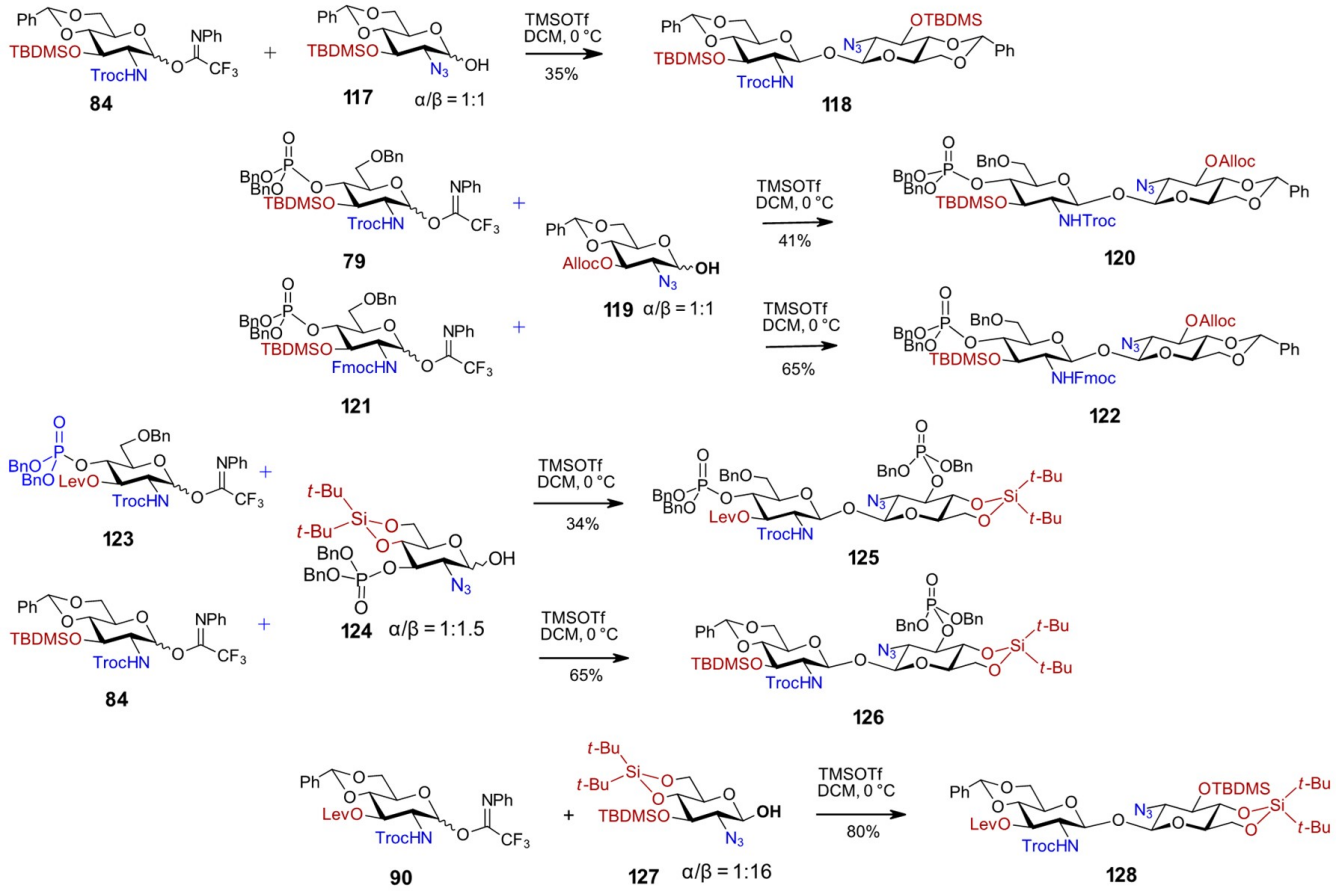
Synthesis of non-symmetric, fully orthogonally protected β,β-1,1'-diglucosamines.

A recent study investigating how acceptor nucleophilicity affects glycosylation selectivity has shown that variations in the type and positioning of functional and protecting groups on the pyranose scaffold can profoundly influence the reaction mechanism, toggling it between S_N_2 and S_N_1 pathways [104–105]. Supporting this, the torsional restriction imparted by the 4,6-*O*-benzylidene group in 2-azido-substituted GlcN lactols was found to favor the formation of the β-anomer during glycosylation [95]. In line with these findings, the glycosylation of the 4,6-*O*-benzylidene-protected 2-azido lactol **117** using imidate donor **84** afforded the symmetrically protected 1,1'-disaccharide **118**, though only in a moderate 35% yield due to the formation of α-configured by-products on the lactol acceptor side (Scheme 10).

Since 2-azido-protected GlcN lactol exists as an α/β = 1:1 anomeric mixture, a strategy was devised to deactivate the thermodynamically favored but less reactive α-anomer. This involved replacing the electron-rich TBDMS group at position 3 with an electron-withdrawing ‘disarming’ substituent, as exemplified by the 3-*O*-Alloc-protected lactol acceptor **119** [95]. The disarming effect of the 4-*O*-phosphate group in the PTFA donor **79** had to be counterbalanced by the electron-donating 3-*O*-TBDMS group, since alternative glycosyl donors bearing temporary electron-withdrawing substituents at position 3 proved insufficiently reactive. It is well-known that the reactivity of glycosyl donors can be significantly enhanced by introducing steric congestion through bulky substituents [99–100,106–108]. When the 3-*O*-TBDMS-protected 2*N*-Troc-protected donor **79** was employed in the glycosylation reaction with anomeric lactol **119** (used in 2-fold excess to compensate for its 50% content of the undesirable α-anomer), the β,β-1,1'-linked disaccharide **120** was obtained, albeit in moderate yield due to the complexity of separating it from reaction components of similar polarity (Scheme 10). Replacing the 2*N*-Troc protecting group with a 2*N*-Fmoc group in the imidate donor **121** led to formation of fully desymmetrized βGlcN(1↔1′)βGlcN disaccharide **122** with an improved isolated yield of 65% [95]. While the “armed” donors **79** and **121** were sufficiently reactive to facilitate the glycosylation of the conformationally and electronically disarmed lactol acceptor **119** (α/β = 1:1), the arming effect of electron-donating substituents also increased the reactivity of the α-anomeric component in lactol **119**, thereby facilitating the formation of diastereomeric by-products [95].

The moderate selectivity observed in glycosylation reactions involving 2-azido-protected GlcN-lactol acceptor **119** was rationalized by the low reactivity of the anomeric center in both the α- and β-configured lactols, due to the disarming influence of the 2-azido group [97,109] and the electronic and torsional disarming effects of the 4,6-*O*-benzylidene acetal [93,110]. Replacing the 4,6-*O*-benzylidene acetal with the less electronically disarming 4,6-*O*-DTBS group, along with the introduction of a disarming phosphate group at the C3–OH, increased the proportion of the β-anomer in lactol **124** (α/β = 2:3) and decreased the reactivity of the α-lactol, thereby restricting glycosylation to the equatorial hydroxy group of the β-configured counterpart [95]. Upon glycosylation of **124** (used in excess; α/β = 2:3) with donor **123**, complete stereoselectivity was observed at the acceptor side, resulting in the exclusive formation of β-configured products, whereas both α- and β-diastereomers were formed on the donor side, ultimately lowering the isolated yield of β,β-1,1'-glycoside **125** to 34% (Scheme 10).

Although donors **79**, **121**, and **123** possess a 1,2-*trans*-directing participating 2*N*-protecting group, glycosylations with low-nucleophilicity β-lactol acceptors still resulted in notable amounts of 1,2-*cis*-linked disaccharides. To mitigate this, incorporation of a 4,6-*O*-benzylidene group proved advantageous, as it is known to significantly decrease the relative reactivity value (RRV) of glucopyranosyl donors, including 2-amino-2-deoxy derivatives, by at least an order of magnitude [48,72,111]. The RRV [72,111] is recognized as a critical factor governing α/β stereoselectivity in NIS/TfOH-promoted glycosylation reactions involving thioglycosides: donors with lower RRVs (disarmed) tend to favor β-anomer formation, while those with higher RRVs (armed) typically promote the formation of α-anomers [112]. Thus, the 4,6-*O*-benzylidene acetal group was introduced to produce torsionally disarmed donors **84** and **90** having apparently lower relative reactivity values. Coupling of the 2-*N*-Troc-protected donor **84** with lactol **124** (α/β = 1:1.5), used in excess to account for its 65% β-anomer content, furnished the β,β-1,1'-linked disaccharide **126** in a 65% yield with full anomeric selectivity (Scheme 10) [113]. Replacement of the phosphate group at C3–OH with an electron-donating TBDMS group, as in lactol **127**, increased the reactivity of the α-anomer, resulting in the formation of undesired α,β-linked diastereomeric by-products, as evidenced by the reaction of **127** (α/β = 1:4) with the imidate donor **90** [103]. However, this specific combination of protecting groups ensured that lactol **127** exhibited a significant shift in the anomeric equilibrium towards the β-anomer (α/β = 1:16), enabling highly efficient glycosylation with the imidate donor **90** to produce the fully orthogonally protected βGlcN(1↔1′)βGlcN product **128** in 80% yield (Scheme 10) [103].

### Synthesis of α,α-1,1'-linked disaccharides

Whereas traditionally used glycosyl donor–acceptor pairs for the construction of α,α-1,1' glycosidic bonds involve benzyl ether- or acetyl-protected monosaccharide lactol acceptors and various types of glycosyl donors, such as thioglycosides, glycosyl imidates, glycosyl bromides and fluorides [51,64], several unconventional approaches, including intramolecular aglycon delivery (IAD), are particularly impressive [114]. As the synthesis of α,α-1,1'-linked disaccharides has already been systematically reviewed [36], particularly with respect to the synthesis of trehalose-containing lipids of mycobacteria [37], this review includes only the most relevant and recent developments in the preparation of α,α-linked 1,1'-glycosides. The review places particular emphasis on the synthesis of unsymmetrical trehalose derivatives, both with respect to the nature of the monosaccharide components (Glc, Gal, Man, GlcN, GalN, etc.) and to the sites of attachment of the protecting and/or functional groups.

**Scheme 11:**
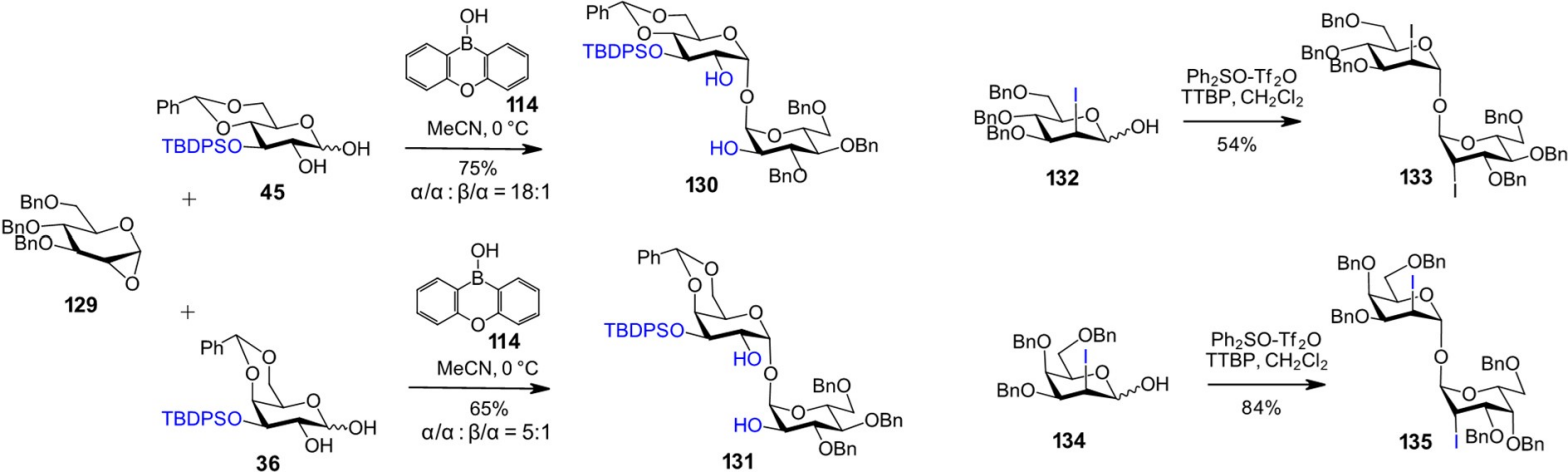
Synthesis of α,α-1,1'-disaccharides.

Over the past decade, several new protocols have been developed for the stereoselective assembly of 1,1'-α,α-disaccharides. For example, a series of α,α-linked 1,1'-disaccharides was obtained using a novel approach that involves the activation of an anhydro sugar glycosyl donor **129** by the "acidic OH group" of a glycosyl 1,2-diol acceptor (generated via activation with a borinic acid catalyst possessing sufficient Lewis acidity) [62–63]. Anomeric C2-OH-unprotected lactols **45** and **36** were subjected to glycosylation with 3,4,6-tri-*O*-benzylated 1,2-anhydroglucose **129** using an organoboron catalyst (Scheme 11). In situ-generated tricyclic borinate complexes (formed between 1,2-glycosyl diols and diarylborinic acids) effectively promoted the glycosylation with glycosyl anhydro sugars, yielding the 1,1'-α,α-trehalose derivatives **130** and **131**, respectively [63]. A separate case of obtaining symmetric 1,1-disaccharides of the *manno*- and *talo*-series **133** and **135**, respectively, via self-condensation of the corresponding 3,4,6-tri-*O*-benzylated 2-iodolactols **132** and **134**, has also been reported (Scheme 11) [115].

**Scheme 12:**
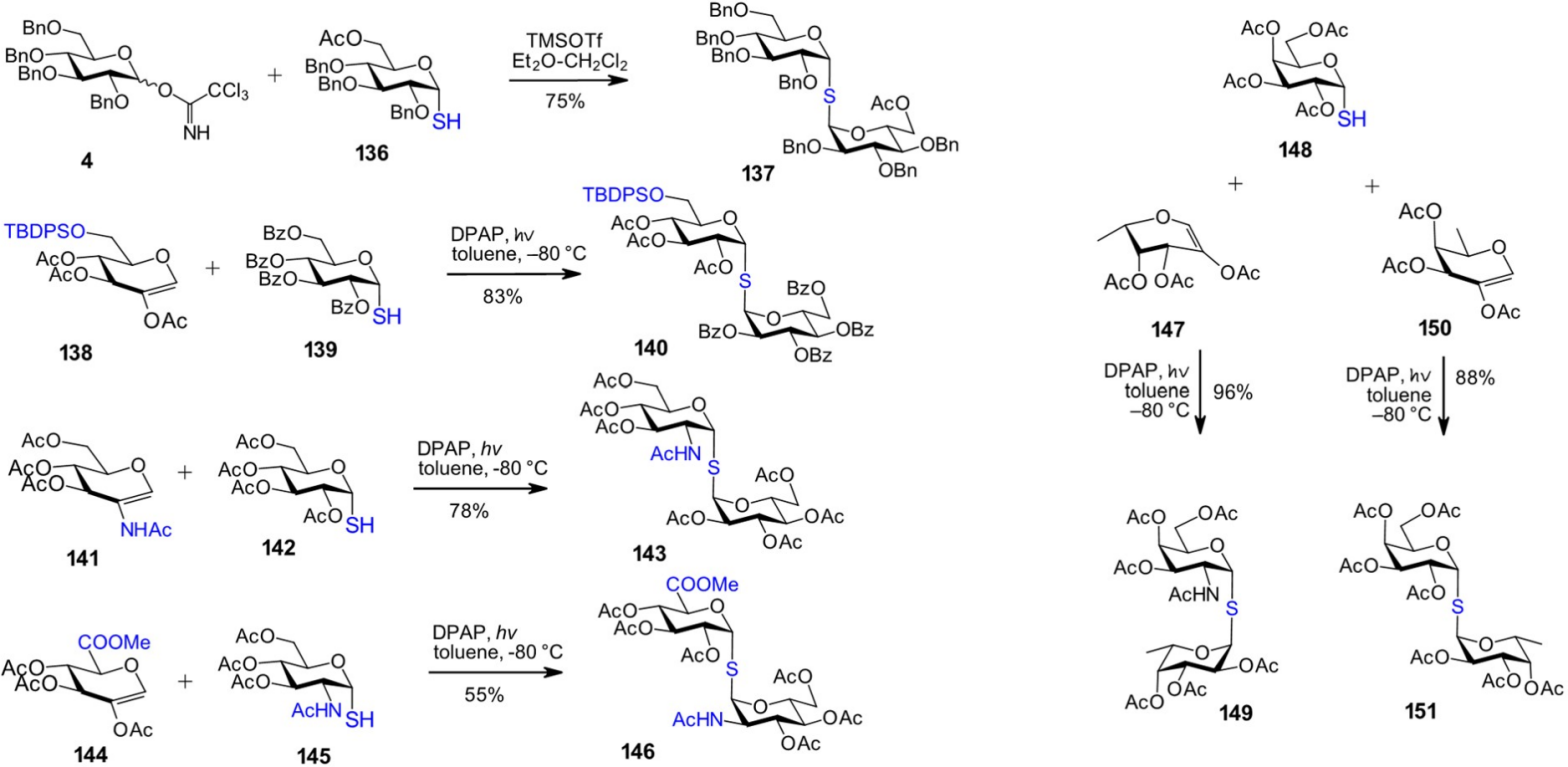
Synthesis of α,α-1,1'-thiodisacchrides.

In specific cases, the chemical synthesis of thiotrehalose analogs [116–117] provides a versatile tool for the construction of therapeutically relevant ligands or inhibitors of carbohydrate-specific innate immune receptors. Synthetic thiodigalactosides and their derivatives have been identified as selective inhibitors of human galectins [118–119], while α,α-1,1'-linked disaccharides composed of a galactose moiety and a second pyranose with varying sugar configurations have been shown to inhibit *Pseudomonas aeruginosa* lectins LecA and LecB [120]. Thioglycosides can be synthesized using standard protocols, typically involving the coupling of an imidate donor with 1-thiols – as exemplified by the reaction of tetrabenzylated trichloroacetimidate donor **4** with an α-configured 1-thiol **136**, yielding thiodiglucoside **137** (Scheme 12).

In addition, novel stereoselective strategies have been developed for the efficient synthesis of α,α-1,1-thioglycosides. 1,2-*cis*-Glycosyl thiols with different sugar configurations were obtained from 2-substituted (mostly 2-acetylated) hexopyranose or 6-deoxyhexopyranose glycals and thioacetic acid under UV irradiation (λ_max_ = 365 nm) using a synergistic photosensitizer–photoinitiator pair: 4-methoxyacetophenone (MAP) and 2,2-dimethoxy-2-phenylacetophenone (DPAP), in a frozen state (−80 °C) with acetic acid as the solvent. A second thiol–ene coupling reaction between the 1,2-*cis*-glycosyl thiols and the 2-substituted glycals produced a series of α,α-1,1'-thiodisaccharides with high stereoselectivity and yield [116]. Along these lines, 6-*O*-silyl-protected triacetyl glycal **138** was reacted with tetrabenzoylated α-thioglucose **139** to provide α,α-1,1'-thiodiglucoside **140** with excellent yield (Scheme 12). 2-Amino-2-deoxypyranoses also served as effective precursors for glycal formation, enabling subsequent thiol–ene coupling reactions with α-glycosyl thiols. For example, GlcNAc-derived glycal **141** was coupled with α-glycosyl thiol **142** to afford α-GlcNAc(1↔1)α-Glc thiodisaccharide **143** [116].

In another instance, glucuronic acid glycal **144** underwent thiol–ene coupling with a GlcNAc-derived α-glycosyl thiol under UV irradiation using DPAP as a cleavable photoinitiator, which gave the thiodisaccharide **146** (Scheme 12) [116]. The success of these reactions was largely attributed to the use of acetic acid as a solvent in its frozen state (−80 °C), combined with stepwise UV exposure (3 × 60 min) at λ_max_ = 365 nm. Using this novel approach, α,α-1,1'-linked Gal-Fuc thiodisaccharides, designed as bispecific ligands for the *Pseudomonas aeruginosa* lectins LecA and LecB, which play key roles in host cell adhesion and exhibit cytotoxic effects, were successfully synthesized [120]. Tetraacetylated α-galactose 1-thiol **148** was reacted with either 2-acetoxy-ʟ-fucal **147** or 2-acetoxy-ᴅ-fucal **150** to afford the thiodisaccharides α-ᴅ-Gal(1↔1)-α-ʟ-Fuc **149** and α-ᴅ-Gal(1↔1)-α-ᴅ-Fuc **151**, respectively (Scheme 12). High yields and excellent stereoselectivity underscore the uniqueness of this specialized protocol for the synthesis of 1,1'-thiodisaccharides.

**Scheme 13:**
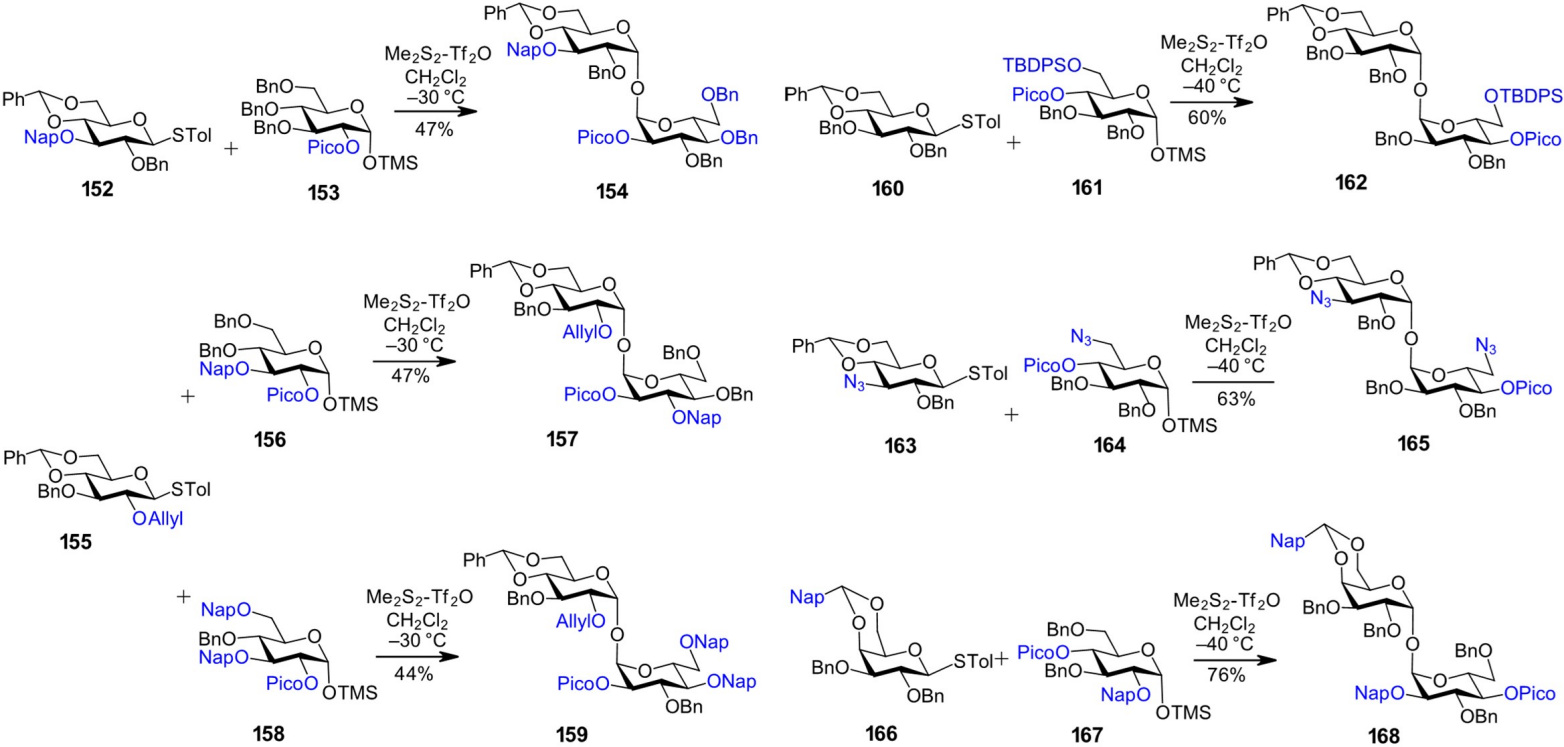
Synthesis of partially desymmetrized α,α-1,1'-linked disaccharides.

Partially desymmetrized trehaloses were obtained from 2-*O*-picoloyl-protected TMS-α-glucosides acting as glycosyl acceptors, in combination with variously protected thioglycoside donors, and were subsequently used for the synthesis of sulfoglycolipids from *M. tuberculosis* [121]. The TMS-glycosides were prepared from the corresponding hemiacetals by acid-catalyzed silylation, involving a silica gel-promoted in situ anomerization of the *O*-TMS-β-glycoside to its more thermodynamically stable α-counterpart [121]. This protocol enabled the efficient synthesis of TMS-protected lactols with a defined anomeric configuration for use as glycosyl acceptors, with the limitation that the protecting groups had to be stable under acidic conditions, therefore, benzyl or 2-naphthylmethyl ethers were used. Glycosylation of the thioglycoside donor **152** with the TMS-protected α-lactol acceptor **153**, using a sulfonium-type promoter prepared from dimethyl disulfide and triflic anhydride, produced the α,α-1,1'-linked diglucoside **154** (Scheme 13) [121]. Similarly, partially desymmetrized trehalose derivatives **157** and **159** were prepared using 2-*O*-picoloyl 3-*O*-Nap- and 3,6-di-*O*-Nap-protected TMS-glycoside acceptors **156** and **158**, respectively (Scheme 13).

The picoloyl protecting group, when placed at the remote C4–OH position, has been shown to additionally stabilize the anomeric configuration in TMS-α-glycoside acceptors, which has been attributed to the formation of a picolinium adduct, formed via the capture of acidic TMSOTf by the pyridine moiety of the picoloyl group [69]. The resulting picolinium-stabilized TMS-glycoside exhibits enhanced configurational stability due to the strong electron-withdrawing nature of the picolinium substituent, which effectively suppresses anomerization. This approach for the preparation of TMS-protected α-lactol acceptors was employed in the synthesis of a series of partially desymmetrized 1,1'-α,α-linked disaccharides (Scheme 13). 6-*O*-TBDPS-protected TMS-α-glucoside **161** was coupled with thioglycoside donor **160**, furnishing the α,α-1,1'-disaccharide **162** with differently protected hydroxy groups at positions 4 and 6 [69]. Additionally, the disaccharide with an azido group at each pyranose moiety **165** was prepared via coupling of the 6-azido-TMS-α-glucoside **164** with the 3-azido-protected donor **163** [71]. Similarly, the nonreducing glucosyl galactoside **168** was synthesized from the thioglycoside donor **166** and the 2-*O*-Nap-protected TMS-α-glucoside acceptor **167** [69].

**Scheme 14:**
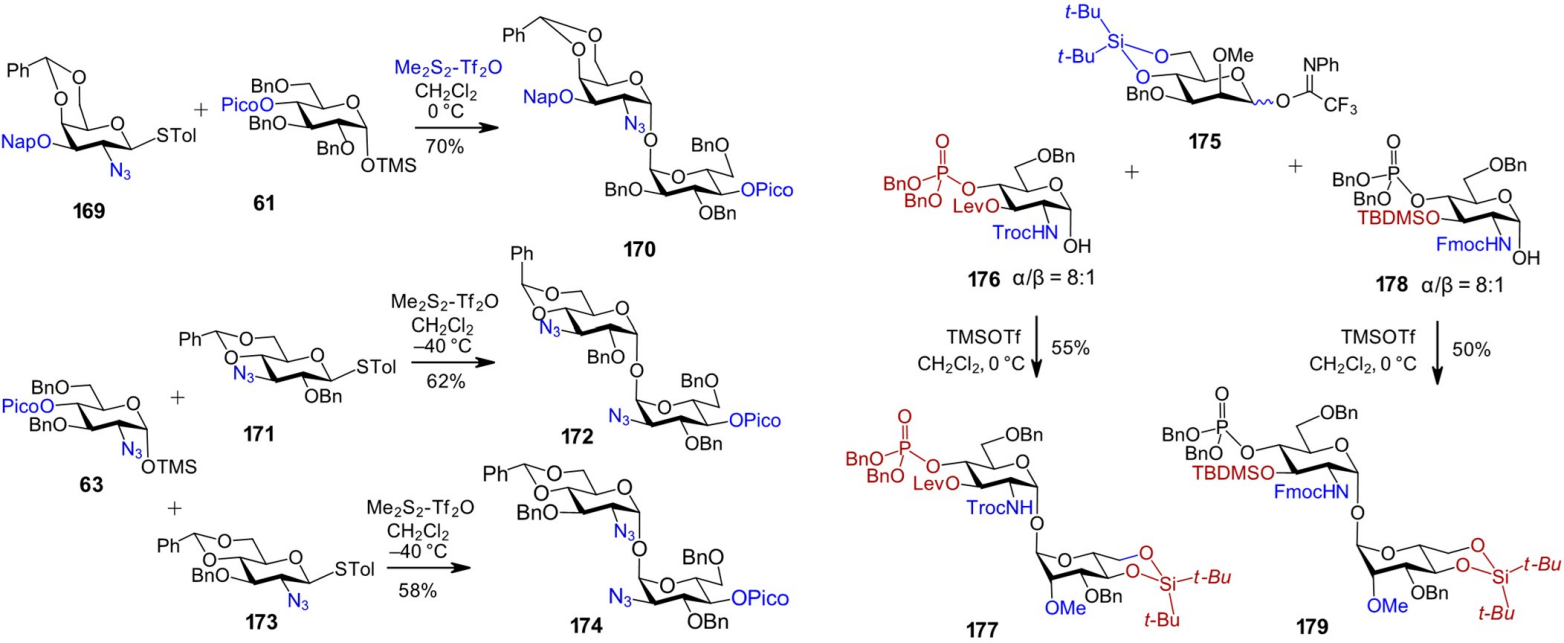
Synthesis of non-symmetric orthogonally protected α,α-1,1'-linked disaccharides involving an aminosugar.

The synthesis of 1,1'-α,α-disaccharides involving 2-amino-2-deoxy sugars presents an even greater challenge, as the range of neighboring protecting groups that favor the formation of α-glycosides is limited, primarily to the 2-azido group, as exemplified by the 3-*O*-Nap-protected thioglycoside donor **169** (Scheme 14). However, in the case of 4,6-*O*-benzylidene acetal-protected GalN, axial stereoselectivity in glycosylation reactions is enhanced, which enabled the successful synthesis of the α-GalN(1↔1')α-Glc disaccharide **170** using the TMS-α-glucoside acceptor **61** [69]. When 2-azido-protected GlcN is used as the glycosyl acceptor, as in the case of α-TMS-glycoside **63**, the α-configuration can still be retained by converting the α-lactol to its corresponding TMS ether, which preserves the anomeric configuration. Subsequent reaction with the thioglycosyl donor **171** afforded the 1,1'-α,α-linked Glc-GlcN conjugate **172** [71]. Similarly, using the 2-azido-protected glycosyl donor **173** in combination with the same GlcN-derived acceptor **63** yielded the 1,1'-α,α-linked diglucosamine **174** (Scheme 14) [69].

When both monosaccharide components of an amino sugar-containing 1,1'-disaccharide require multiple orthogonal protecting or functional groups, the use of a picoloyl group at position 4 or 2 to stabilize the anomeric configuration is not feasible. In such cases, the structure of the lactol acceptor typically relies on 2*N*-carbamate-protected GlcN derivatives, such as the 2*N*-Troc-protected lactol **176** or the 2*N*-Fmoc-protected lactol **178**, in which the α-configuration is reinforced by a hydrogen bond between the anomeric OH group and the *N*-carbamate moiety (Scheme 14). The α-anomeric configuration on the side of the imidate glycosyl donor can be controlled through the use of neighboring, participating protecting groups, as exemplified by the 4,6-*O*-DTBS-protected donor **175** [122]. Using this strategy, two α-GlcN(1↔1')α-Man disaccharides **177** and **179** bearing multiple orthogonal protecting groups were synthesized via TMSOTf-promoted glycosylation reactions with α-lactol acceptors **176** and **178**, respectively [123]. These nonreducing disaccharides were employed as scaffolds for the synthesis of immunomodulatory glycolipids – TLR4 agonists with picomolar potency [124].

## Conclusion

Approaches to 1,1'-glycosylation can be rationally selected based on the required anomeric configuration and the number and position of the functional groups in the target nonreducing disaccharide-containing biomolecule. While some methodologies offer excellent stereoselectivity and yields, they impose certain restrictions on the choice of protecting groups, particularly with regard to their type and positioning on the monosaccharide units. Other protocols allow for the preparation of highly desymmetrized 1,1'-disaccharides, albeit sometimes at the expense of only moderate isolated yields. Achieving stereoselectivity on the lactol acceptor side remains a critical challenge, for which several strategies have been developed. Some approaches employ specialized reagents to lock the anomeric hydroxy group in an axial or equatorial orientation, while others rely on the strategic placement of specific protecting groups at remote or neighboring positions.

Several recommendations for achieving stereoselective 1,1'-glycosylation have emerged, particularly through the tuning of reactivity via protecting group strategies. In particular, the inclusion of 4,6-*O*-cyclic protecting groups in both the glycosyl donor and the lactol acceptor has proven beneficial, as this modification enhances stereoselectivity in 1,1'-glycosylation reactions by exerting a torsional disarming effect. In line with the concept of matching the nucleophilicity of lactol acceptors with the reactivity of glycosyl donors, the combination of 4,6-*O*-DTBS-tethered lactols as acceptors and 4,6-*O*-benzylidene-2-*N*-Troc-protected glycosyl imidates as donors has proven particularly effective for the stereoselective synthesis of fully orthogonally protected β,α- and β,β-1,1'-disaccharides. A consistent pattern of anomeric preference was observed among GlcN-derived lactol acceptors: the 2-*N*-carbamate protecting groups contributed to the stabilization of the α-anomer – an effect further reinforced by the torsional constraints imposed by the 4,6-*O*-cyclic protecting groups.

The β-anomeric lactols were found to be more stabilized in 2-azido-protected GlcN derivatives, where the reactivity of α- and β-configured lactols can be selectively modulated by introducing specific electron-withdrawing groups at remote positions. These groups reduce the nucleophilicity of the α-lactol component, thereby enabling the selective participation of the β-lactol in glycosylation reactions. Previous studies have shown that, being inherently less nucleophilic, secondary hydroxy groups in glycosyl acceptors tend to favor α-glycoside formation – a trend that becomes more pronounced with decreasing acceptor reactivity [105,125–126]. This principle also applies to modulating the α/β ratio in lactol acceptors: the introduction of electron-withdrawing groups at remote positions shifts the equilibrium towards the α-lactol, whereas electron-donating substituents increase the proportion of the β-anomer, thereby improving glycosylation stereoselectivity.

## Data Availability

Data sharing is not applicable as no new data was generated or analyzed in this study.
